# Supplementary figures and images for: A novel class of Candida glabrata cell wall proteins with β-helix fold mediates adhesion in clinical isolates
Source: PLoS Pathog. 2021 Dec 28;17(12):e1009980. doi: 10.1371/journal.ppat.1009980 (PMC8746771; doi:10.1371/journal.ppat.1009980)

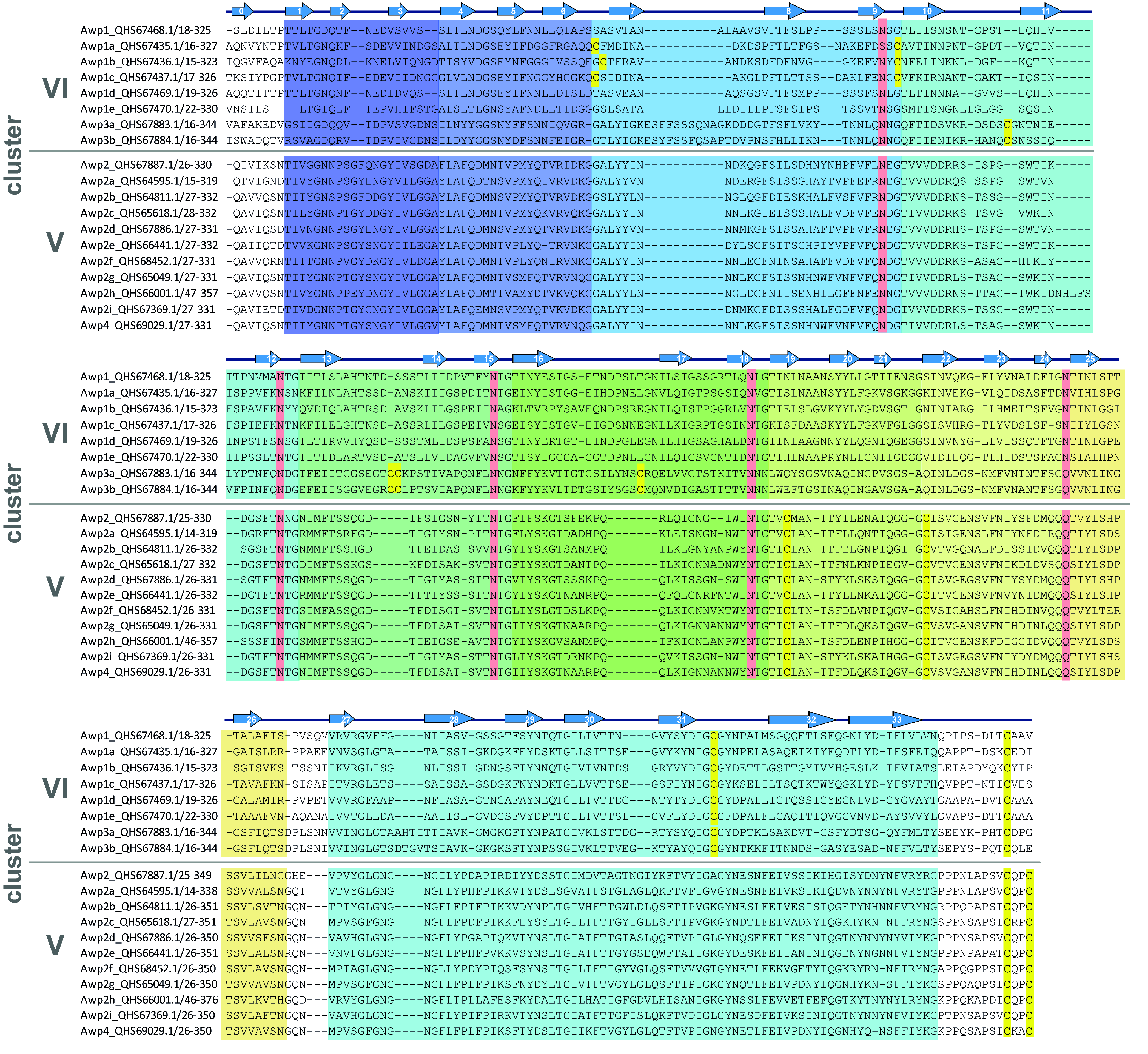

Supplement: S1 Fig — The MSA was generated for A-regions of C. glabrata GPI-CWPs with β-helix domains by 3D-coffee using the structural information of the Awp1-A and Awp3b-A domains. The nine β-helix turns are highlighted by blue-to-yellow coloured boxes; the α-crystalline domain by a cyan box. Cysteines are highlighted in yellow. Notably, pairwise sequence identities of Awp2, Awp2a-Awp2i and Awp4 A-regions vary from 53.6% to 94.8% (cluster V), those of Awp1,Awp1a-Awp1e, Awp3a and Awp3b (cluster VI) from 18.1% to 64.1%. For clarity, distantly related Awp1-like adhesins of cluster V (QHS65613.1, QHS67215.1, QHS68879.1) are omitted from the MSA. Anyway, pairwise sequence identities between clusters V and VI A-regions are low, i.e. in the range of 14.4% to 22.9%. (TIF) [file ppat.1009980.s006.tif]

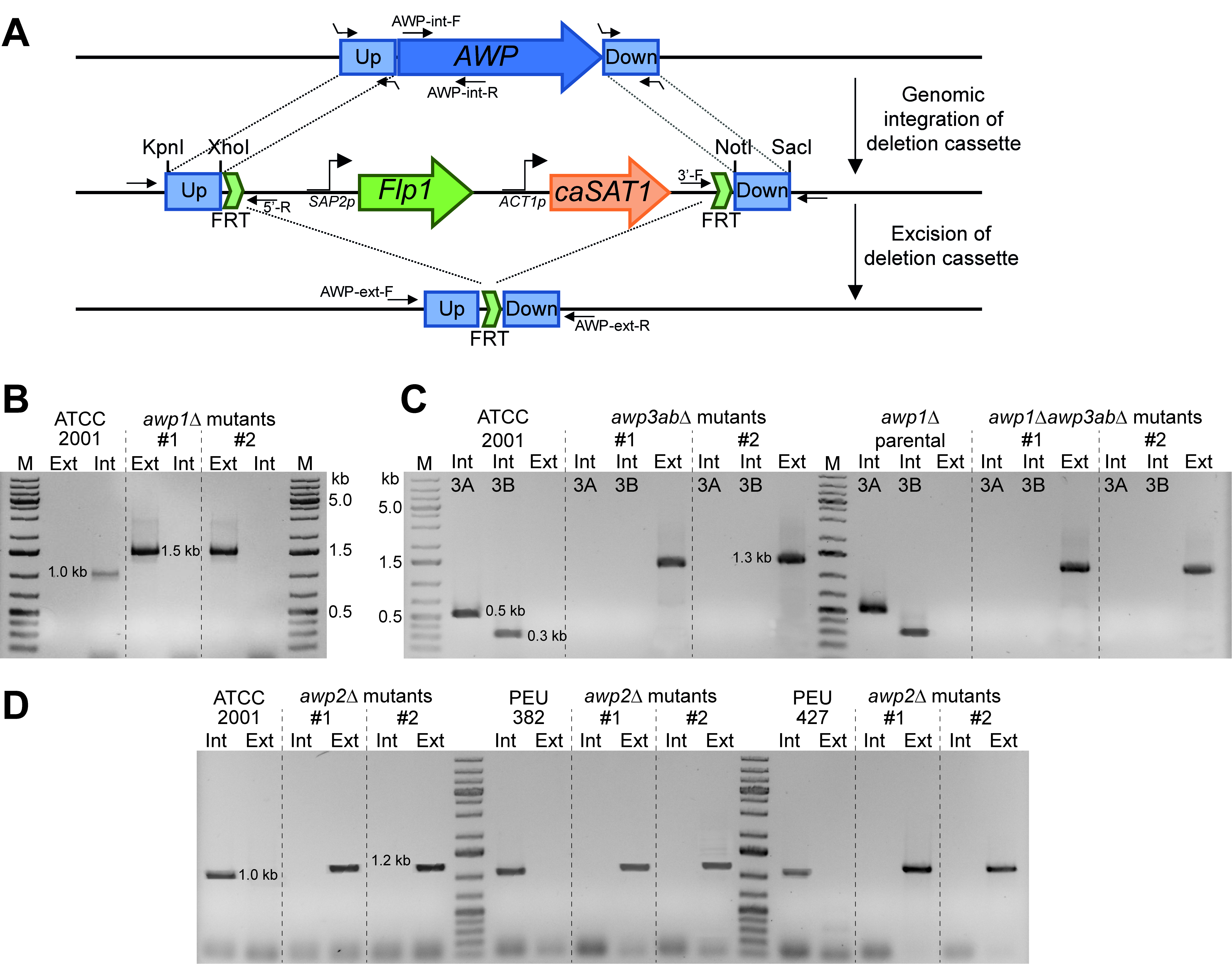

Supplement: S2 Fig — (A) Schematic structure of SAT1-flipping deletion constructs and AWP gene deletion procedure. CRISPR-Cas9 RNP complexes (not indicated) are added during transformation to aid integration into the correct locus. Mutants are selected by PCR analysis of 5’ and 3’ integration junctions, after which the cassette is excised by inducing FLP1 expression. (B-D). PCR verification of AWP deletion mutants after excision of the cassette using external (Ext) and internal (Int) primers as indicated in (A). (B) Verification of awp1Δ mutants. (C) Verification of awp3abΔ mutants and awp1Δ /awp3abΔ mutants. (D) Verification of awp2Δ mutants in three different genetic backgrounds. (TIF) [file ppat.1009980.s007.tif]

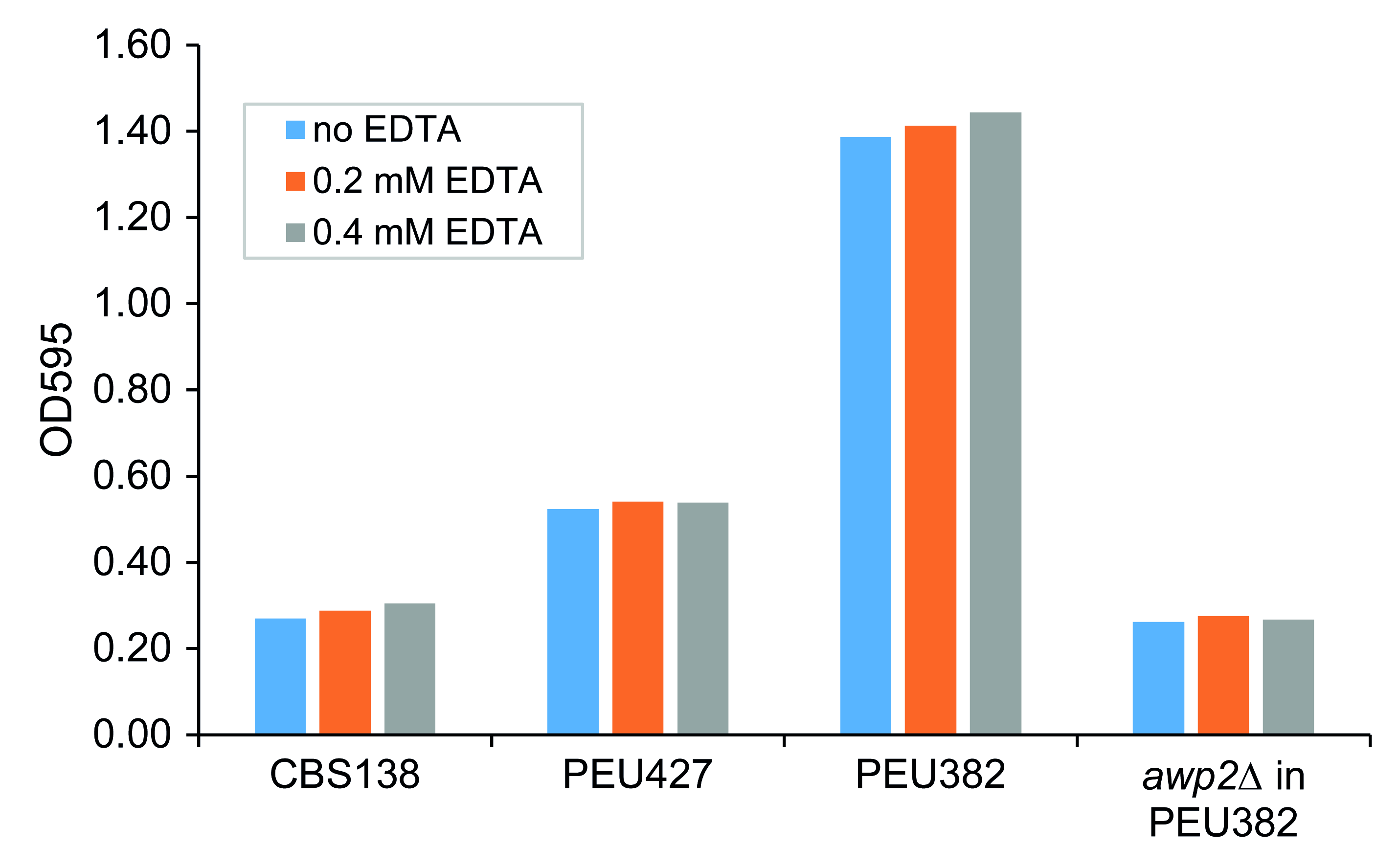

Supplement: S3 Fig — Adhesion to polystyrene after 24 h of incubation in YPD in the presence of the indicated EDTA concentrations. Notably, the MIC50 for all four tested strains is 0.8 mM. (TIF) [file ppat.1009980.s008.tif]

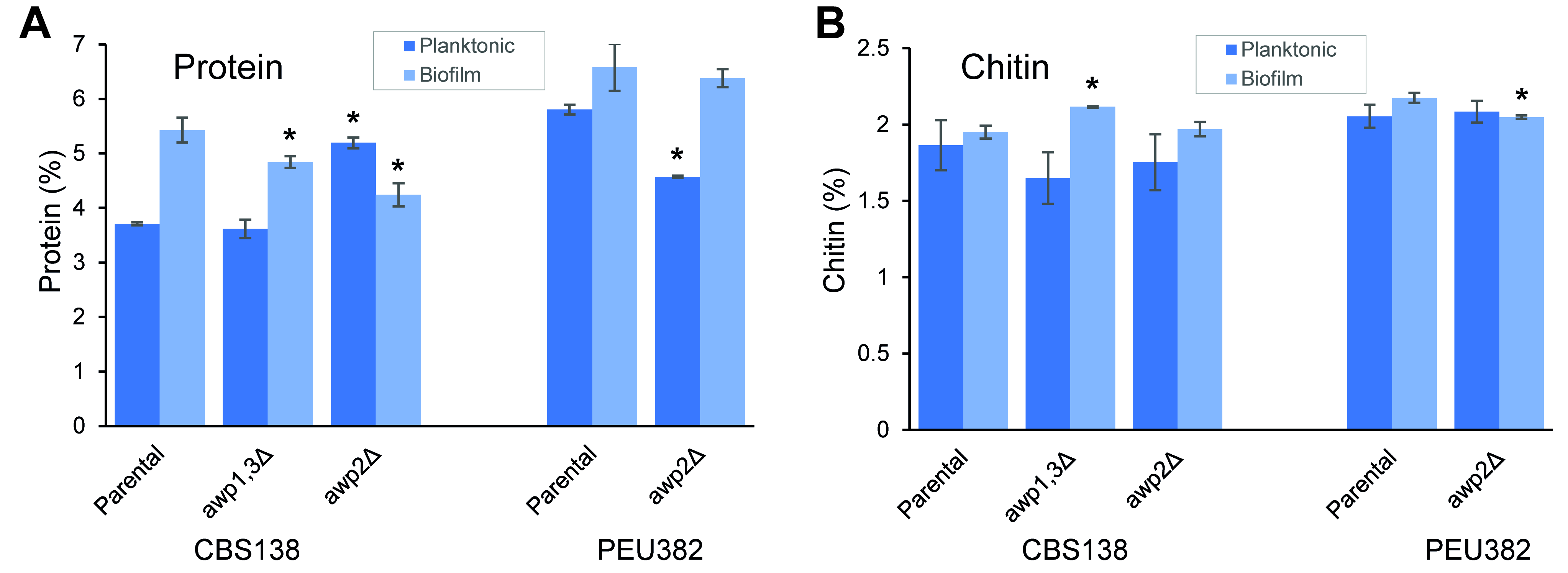

Supplement: S4 Fig — Protein and chitin amount in the cell wall was measured using colorimetric assays as described in Kapteyn et al. (2001). Each strain was assayed twice with two technical replicates each. Statistically significant differences (Student’s t-tests or ANOVA, p<0.05) of mutants compared to their parental strain under the same conditions are indicated by asterisks. (TIF) [file ppat.1009980.s009.tif]

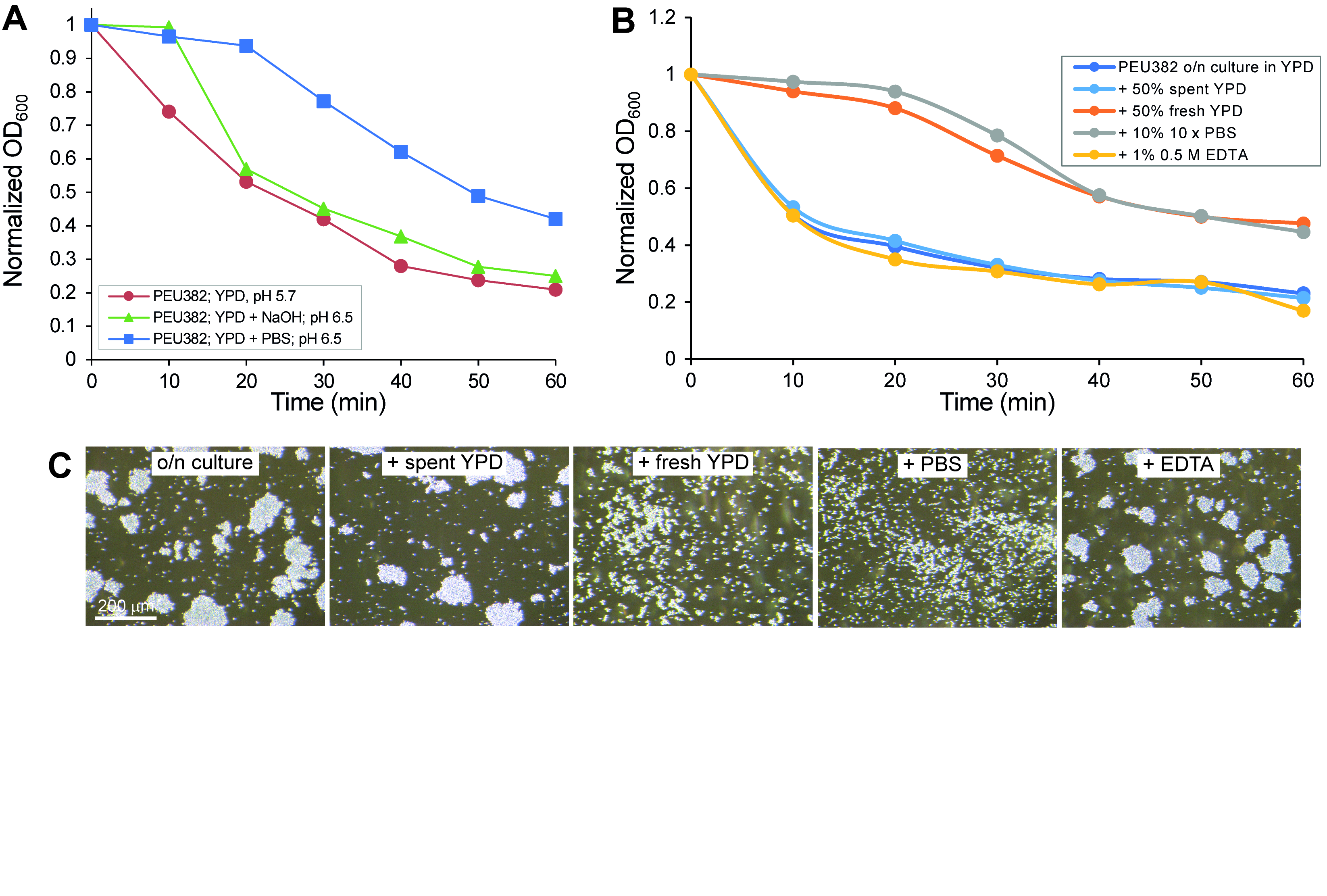

Supplement: S5 Fig — (A) Sedimentation of a 37°C overnight culture of hyperadhesive aggregating strain PEU382 in YPD with and without pH adjustment by adding PBS (final conc. 1×) or NaOH was followed in time. (B) Sedimentation of PEU382 cells grown overnight at 37°C in YPD without or with supplementations as indicated. (C) Microscopical images of cells from (B) at t = 0. (TIF) [file ppat.1009980.s010.tif]

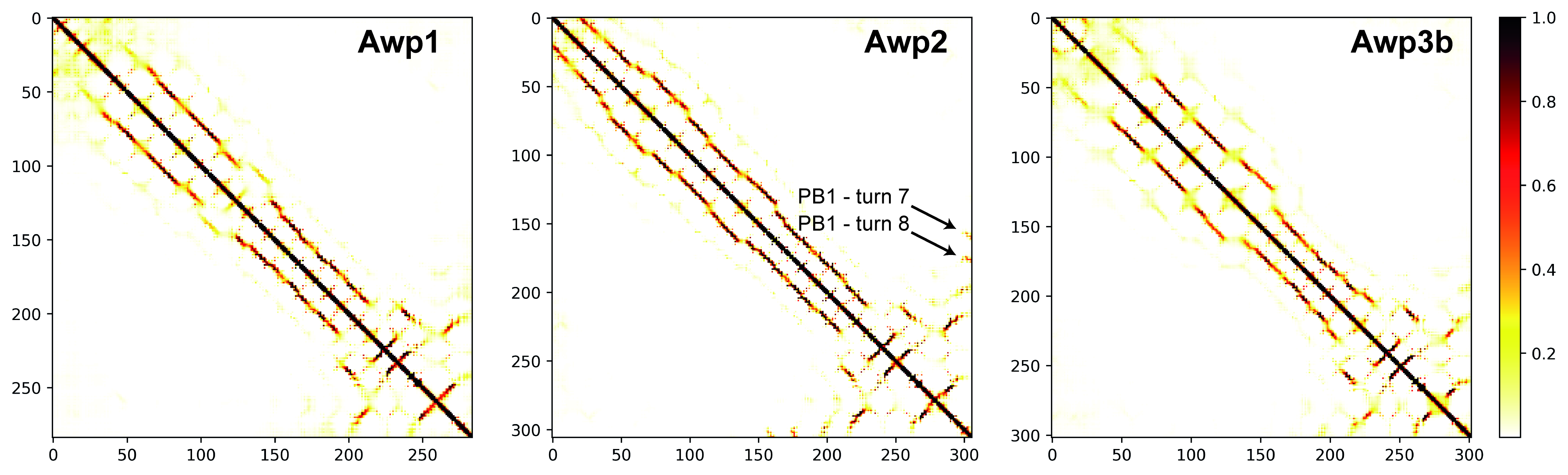

Supplement: S6 Fig — Contact maps were predicted by trRosetta [19] using therewith derived multiple sequence alignments of 532, 195 and 140 homologous sequences for Awp1, Awp2, and Awp3b, respectively. The TM-scores of the obtained models were very high with values of 0.738–0.839. Predicted contacts between the C-terminal end of the Awp2 A-region and β-helix turns 7 and 8 are highlighted by arrows. These contacts are the base for the disulfide bridges C327-C201 and C330-C184 in the Awp2 model. (TIF) [file ppat.1009980.s011.tif]

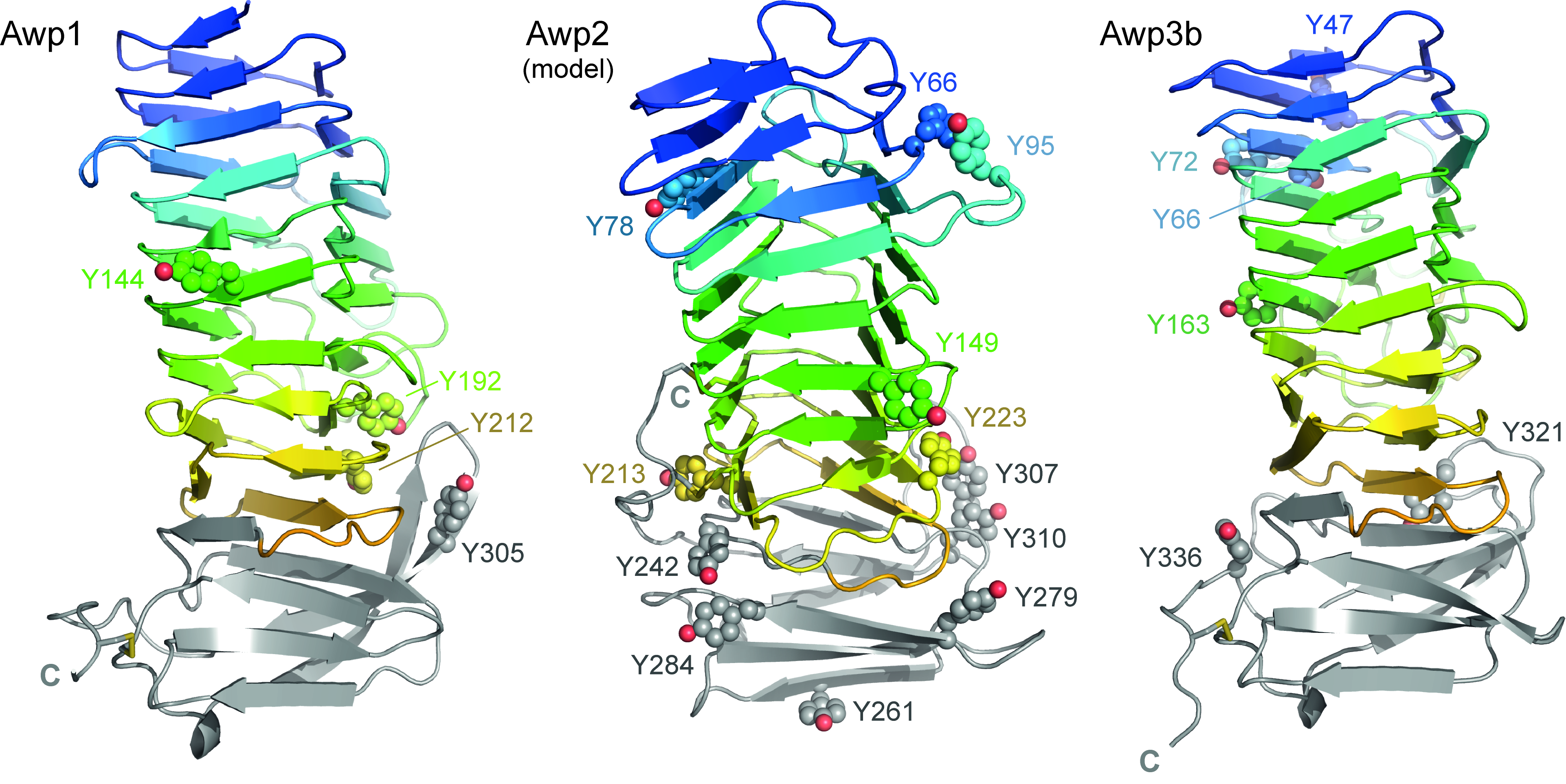

Supplement: S7 Fig — Only tyrosines have been found to be surface-exposed on Awp1-related A-regions. Awp2 harbors six residues each in the β-helix (coloured) and α-crystallin (gray) domain. (TIF) [file ppat.1009980.s012.tif]
