# Supplementary material for: A novel class of Candida glabrata cell wall proteins with β-helix fold mediates adhesion in clinical isolates
Source: PLoS Pathog. 2021 Dec 28;17(12):e1009980. doi: 10.1371/journal.ppat.1009980 (PMC8746771; doi:10.1371/journal.ppat.1009980)
Supplement: Attachment — Submitted filename: 20211118_RevisionRequests_answers.docx [file ppat.1009980.s013.docx]

**Part II – Major Issues: Key Experiments Required for Acceptance**

Reviewer #1: There are a few questions or concerns that should be addressed:
Q1: One of my major questions had to do with the possible role of metal cations in the function of these adhesins (particularly Awp3b). The Gd3+-soaked crystals used for phase determination showed an impressive array of Gd3+ ions, most of which were not directly coordinated by surface Awp3b residues. However, several of these Gd3+ ions were surface-bound; given the common use of lanthanides to replace Ca2+ ions in specific sites on proteins, it is an intriguing finding. I suspect the surface-bound Gd3+ positions may represent low-affinity metal binding sites for Ca2+ or another metal cation.

A1: The referee is right when noting that surface-bound Gd3+ ions may mimic low-affinity metal binding sites for calcium and related ions. Previously, for the A-domain of Flo5, a C-type lectin in *S. cerevisiae* that binds non-reducing Man-α1,2-Man ends, we have noted that Gd3+ indeed occupies the conserved calcium binding site that is required for carbohydrate binding (Veelders et al., 2010). However, our subsequent studies on the formation of larger-scale gadolinium clusters along the surface region of Flo5 (Veelders et al., 2012) showed that other Gd3+-binding sites were not at all related to the primary and secondary carbohydrate binding site in Flo5. As we further found no evidence that calcium binding affects the biological function of Awp1-related adhesins (Q2, Q6), we may have been a bit too brief about this issue in the previous version of the manuscript. See also our answers to Q2 and Q6.

Q2: In terms of the potential metal binding sites on Awp3b, were any computational approaches used to identify likely metal binding sites in the structure? There are various programs or servers mentioned in the literature for this type of analysis such as CheckMyMetal (<http://csgid.org/csgid/metal_sites/>). It would be a good idea to use this server or a similar program to see whether the surface-bound Gd3+ are consistent with a bona fide binding site for Ca2+ or another metal ion. Was any electron density visible in the native Awp3b (7O9O) structure at the locations where Gd3+ ions were observed at the protein surface in the phased structure (7O9Q)?

A2: The crystal structure of native Awp3b showed no suspicious electron density at the Gd3+-binding sites that may be attributed to other cations. Given the referee’s interest for some details about our observed Gd3+ binding sites, we added the following to the last paragraph of the discussion. “… *Most residues liganding to Gd3+ ions like D40 and E59, which coordinate the Gd21-cluster 1, or Q102, E132, and E134, which bind to the tetranuclear Gd3+ cluster 2 (Figure 7B) are not conserved among Awp1-related A-domains. An analysis of the Awp3b-A•Gd3+ complex by the CMM server (37) shows that only the binding site for Gd3+ cluster 3, which involves the side chains of E141, D169 and the carbonyl groups of K109 and R110, fulfills the geometric criteria expected for a mononuclear Ca2+-binding site. Accordingly*, a structural alignment with the pectate lyase C … “

Q3: The authors mention at the top of page 5 that washing (or diluting) the PEU382 cells with PBS abolished hyperaggregation and typical sedimentation behavior, which could potentially be due to the loss of surface-bound Ca2+ (or other) metal ions, resulting in loss of intercellular adhesion. Although dilution with NaOH instead of PBS failed to show the same effect, phosphate is known to cause solubility issues with many divalent cations such as Ca2+, so the PBS effect could still be responsible for metal loss. Therefore, I would strongly recommend conducting hyperaggregation, sedimentation, and adhesion assays by adding a strong chelator such as DTPA or EDTA to remove any bioavailable metal cations to see if the adhesive functions are lost; then various metal cations can be added back to a minimal inhibitory concentration of chelator to check for restoration of the adhesive function. These experiments can also be done using chemically defined media, but the chelator experiment is usually technically more feasible. Even low-affinity metal binding sites could certainly modulate the adhesive properties of these Awp proteins.

A3: The referee is right in noting that calcium and other bivalent cations might have a strong effect in adhesion; *S. cerevisiae* flocculins like Flo1 and Flo5 represent exquisite examples for this mode of adhesive function. We performed adhesion experiments in the presence of EDTA to check this (shown now as novel Supplementary Figure S3) and found no effect, indicating that bivalent cations like calcium or magnesium do not affect the adhesion behavior of our *C. glabrata* strains to plastics at all. We added the following to the Results section: “…diminished adherence to a level similar to the reference strain. *Awp2-mediated adhesion of C. glabrata to polystyrene is independent of calcium and other bivalent cations, as addition of EDTA to the assays caused no loss of strain-specific adhesion (Supplementary Figure S3).* …”

Q4: The authors mentioned that the proteins were submitted for glycan array screening but that no glycan ligands were identified. What buffer was used for the glycan array screening? If there is a requirement for metal ions for adhesive function (as is often the case with protein-glycan interactions), potential ligands could be missed using a minimal buffer without the necessary metals.

A4: The referee is right in being cautious about CFG conditions. However, we didn’t find any functional nor structural evidence that prompted us to include calcium or other bivalent cations in glycan arrays (see A2, A3). Hence, the material has been supplied to CFG in the size-exclusion buffer without added metal ions.

Q5:The exterior stacking of residues on the surface of the Awp structures is very interesting and reminiscent of bacterial β-helical ice-binding proteins, which form a flat planar protein surface that will form periodic H-bonds with the surface of ice crystals (see for example, Mangiagalli et al., doi: 10.1111/febs.14434; or Guo et al., doi: 10.1126/sciadv.1701440). Although this would not be relevant to the binding of hydrophobic polystyrene surfaces, it might be relevant to adhesion to other abiotic hydrophilic surfaces such as glass. Does C. glabrata adhere to both polystyrene and glass surfaces? Do the residues in these exterior stacking interactions form a flat protein surface that might interact with a planar abiotic substrate? Consistent with this idea, it’s interesting that very few (or zero) predicted O-glycosylation sites were identified in the β-helix subdomains, given that glycans would sterically inhibit interaction with an abiotic surface.

A5: Thanks a lot for the wonderful suggestion by the referee. We checked the adhesion of our *C. glabrata* strains to glass. Interestingly, we found that adhesion to glass is diminished for PEU382 but not PEU382/*awp2*Δ when compared to the other CBS138 and PEU427 strains. This result implies an anti-adhesive function of Awp2 to glass at least in the PEU382 context, which may be rationalized by its contribution to the hydrophobicity of the PEU382 strain.

We ADDED to Results: “*A dominant role of Awp2 for causing the hydrophobic surface character of the PEU382 strain can be also inferred from the twofold diminished adhesion to hydrophilic glass surfaces by PEU382 when compared to CBS138 and PEU427 (Figure 2C). Deletion of AWP2 in PEU382 restores the adhesion to glass surfaces to even higher levels than exercised by CBS138 and PEU427, indicating that at least some Awp1-related A-regions have anti-adhesive functions as well*.”

Reviewer #2:

Q6: Gd++ binding and the effects of a PBS wash strongly suggest cation binding is key to activity. This point needs to be clarified throughout. PBS was used in the 4 hr adhesions, but not the 24 hr adhesions. Thus, effects of PBS, neutral pH and cation binding in Awp2-expressing cells (and also Awp3-expressing ?) are critical questions. Adhesion experiments at neutral pH w/wo EDTA and added cations would help point us in the right direction.

A6: The referee is right in his/her request as Referee 1 also did in Q2 and Q3. The suggested adhesion experiments in the presence of EDTA have been performed (A3) and ruled out a role for bivalent cations in mediating *C. glabrata* adhesiveness to plastics. Initially, we also had in mind that the PBS effect might be due to the phosphate content of this buffer system and some interference with bivalent cations. However, this is not the case as we observed the same effect by adding fresh YPD media, but not EDTA. The following was added to the Results section.

“… but not when NaOH was added to reach the same pH 6.5. *Interestingly, the same effect could be achieved by adding fresh YPD media, but not by EDTA or YPD media conditioned before by C. glabrata growth therein (Supplementary Figures S5B, S5C). These results suggest that there may other factors than phosphate, which may inhibit hyperaggregation of PEU382.* Nevertheless, …”

Reviewer #3:

Q7: The authors used Hela cells to determine the contribution to adhesion to human cells. The gene and protein expression of Hela cells vary from lab to lab (PMID: 30778230). Furthermore, these cells are derived from a cancerous cervical tumor and are not reflective of a normal barrier cell (epithelial or endothelial cell). Given that the authors should consider primary human cells.

A7: The referee is right in noting that this cervical cell line is only of limited use when considering the potential biomedical aspect of *C. glabrata* adhesion. Accordingly, we repeated the adhesion assays with HaCaT cells, which represent a human, immortalized keratinocyte cell line. These data are now presented in a new Table 1, which compares the adhesion of our *C. glabrata* strains to these two human cell lines. The results again showed that there is no role of Awp1-Awp3 in mediating adhesion to human cels, beit HaCaT or HeLa cells. Notably, this set of experiments showed a clear preference of the clinical isolate PEU427 for adherence to these human model cell lines, especially to HaCaT.

**Part III – Minor Issues: Editorial and Data Presentation Modifications**

Reviewer #1:

Q8: On page 4, 2nd line of final paragraph: the abbreviations CFW, CR, and SDS are used without prior explanation. Although SDS is fairly standard, CFW and CR are not and should be spelled out the first time they are used.

A8: Thanks, corrected, and we actually removed most of the superfluous abbreviations by replacing with full names

Q9: In the final paragraph on page 6, a series of residues that form a Ser/Thr ladder are described, with a reference to Figure 6 (“…S45, S65, T92, T120, T148…”). It would be very helpful to label (at least some of) these residues in Fig 6, since the residues forming the outer stack in the +2 residue position are already labeled and it’s confusing to then look through all the labels and not see those listed for the Ser/Thr ladder.

A9: The referee is right. “S45” was wrong and corrected to “S42”. The requested labels have been added to Figure 6A.

Q10: The PDB validation reports listed several RSRZ outliers in a couple of the structures, but this analysis is known to be rather unreliable by the crystallography community. To get a better validation report, please run the structures through the MolProbity server and report the ClashScore and percentiles in Table 1. (Other than the questionable RSRZ values, the crystal structures appeared to be well refined).

A10: Thanks a lot for the comment, the referee is right. Actually, the (now) Table 2 lists the geometric data as produced by the PHENIX implementation of MolProbity. We added a note about that to Table 2 and included the missing ClashScore.

Reviewer #2:

Q11 & A11: Abstract
1. Define ‘clusters’: are these homologous clusters, clusters of chromosomal loci, or both?

CORRECTED: “homology clusters”

2. “Awp3b-A” meaning is not clear. Should it be “The A domain of Awp3b (Awp3b-A)…”?

CORRECTED: “The A-region of Awp3b”

3. Why is Gd binding important? Please see #16 below on cation binding.

A: Gadolinium binding is not per se important in the context of our analysis of Awp1-related adhesins. It is the sheer size of the observed cluster!

4. “…was observed and phenotypic...” is run-on. I suggest “…was observed. Phenotypic...”

CORRECTED as suggested

5. “…for adhesiveness, as well as..” I suggest “…for adhesion to polystyrene, as well as…”

CORRECTED as suggested

6. The sentence “In contrast, deletion…” is long and hard to read.

SHORTEND as suggested

Q12 & A12: Intro
p.2, paragraph 1
7. Define ‘clusters’ Are there physically linked clusters, or just sequence-similar? See #1

ADDED “sequence” in …. “studies that – based on A-region *sequence* similarity – were categorized into seven …”

8. “Although phylogenetically being more

CHANGED to “Despite a closer relationship to *Saccharomyces* than to the pathogenic CTG-clade *Candida* spp. …”

9. “..effector domains, or functional domains –“

DELETED

10. “By being linked with its processed C-terminus to cell wall β-1,6-glucans via the glycan remnant of the GPI anchor, the heavily glycosylated B-region can interact with other cell wall glycans and act as spacer molecule to breach through the wall to present the A-region along the cell surface (1, 6).” Here and Fig. 8: it is likely that the modified GPI’s are linked to the outer layers of wall glucan, and so may not need to ‘breach through the wall’.

That is actually what was initially intended in this sentence. The GPI-CWP is covalently linked to the cell wall via the 1,6-glucans. DELETED “to breach through the wall”. Figure 8B is adjusted as in #32

11. Paragraph 2 “in other fungi, which may raise”[suggest “a finding which raises”]

CHANGED as suggested

12. Paragraph 3 planktonic growth (4),” specify which strain

ADDED as suggested, CBS138

13. Paragraph 5 “Phylogenetic analysis revealed weak structural relationships” [suggest “Sequence analysis revealed weak similarities leading to predictions of structural similarities...”

Thanks, CHANGED as suggested

14. Paragraph 1 “…S. cerevisiae harbors only two, Hpf1 and Css1.” S. cerevisiae Awa1 mentioned is mentioned later as a homolog, but is not listed here

The referee is right. Indeed, Awa1 is not listed here because it only occurs in a few S. c. strains like the Sake strain Kyokai 7. We hence ADDED “S288c”

15. Paragraph 2 In Candida glabrata CBS138 we find [found] 22 paralogs of Awp1

CHANGED as suggested

16. Gd++ binding and the effects of a PBS wash strongly suggest cation binding is key to activity. This point needs to be clarified throughout. PBS was used in the 4 hr adhesions, but not the 24 hr adhesions. Thus, effects of PBS, neutral pH and cation binding in Awp2-expressing cells (and also Awp3-expressing ?) are critical questions. Adhesion experiments at neutral pH w/wo EDTA and added cations would help point us in the right direction.

These points are already addressed in A3 and A6.

17. “…PEU427 and CBS138 and awp1,3Δ double mutants…” should be “..PEU427 and CBS138, because awp1,3Δ…”

The sentence was correct, but may have been misunderstood. We CHANGED it to “Contrasting with the many surface phenotypes of *awp2Δ* in PEU382, *awp2Δ* mutants in PEU427 and CBS138 as well as *awp1,3Δ* double mutants in CBS138 lacked obvious cell surface-related phenotypes as only minor alter”

18. Paragraph 2 “The roles of the Ser/Thr and the Asn stacks on the surfaces Awp1-related A-domain β-helices hence remain elusive.” See Richardson, J. S. (1981) The anatomy and taxonomy of protein structure. Adv. Protein Chem. Biochem. 35, 167–339. This review clearly predicts the roles of ASN in the β-helical turns

Thanks a lot. This is a fundamental review highly beloved during my study times. Thanks again! ADDED as “domain β-helices *apart from structural stabilization of the β-helical turns (24)* hence remain”

19. “RMSD values ranging from 2.63 to 6.04 Å” --In this reviewer’s opinion, 6 Å is a characteristic of structural similarity, but not ‘high structural similarity’

The referee is right, CORRECTED

20. The analyses seem to predict that Awp2 bind divalent cations and glycans. Cellular adhesion assays with EDTA and divalents will clarify this point. See #3, #16

See A3 and A6!

Discussion
21. Paragraph 4. The discussion of cell wall association implies membrane binding and penetration through the wall. This is probably incorrect, according to previous publications by one of the authors. See doi: 10.1128/EC.00284-08.

The referee is right. This paragraph may have been easily misunderstood. We adjusted it including the illustrating Figure 8B.

22. Paragraph 7. “residues (18, 25)” There is a recent highly relevant reference: doi: 10.7554/eLife.6859

Thanks, ADDED

M&M
Generation of deletion mutants
23. line 2: (12)_ whereas

DONE

Legends
24. Fig. 1: Title: should be “SSN analysis…

DONE

25. The edge inclusion criteria in the L and R graphics should be specified.

DONE

26. Add an explanation of the coloring, e.g.: “The nodes are colored by fungal clade, with Red being C. glabrata (Nakaseomyces). Light orange designated the CTG clade, including Candida albicans.”

ADDED as suggested

Fig. 2
27. Define FC

ADDED “flow cytometry”

28. Correct to: “Data in (B)[A] and (C) are normalized to CBS138…”

DONE. Please note that 3C moved to Table 1.

Fig. 3
29. 3D: Add “200 ug/ml calcofluor white” to Y axis label

DONE

30. 3F top panels need better contrast

DONE. We have provided better quality images.

31. Fig. 6; Awp 2 is not shown and should be omitted from the title

A. Thanks, DONE

32. Fig. 8 Alter the CW attachment cartoon to acknowledge attachment to glucan. See #10 and #21.A. Thanks for the comment to make the cartoon a bit better understandable. We introduced proper icons for the C-region and the GPI-remnant. To avoid misunderstandings, we added labels for the β-1,6/ β-1,3-glucan layer.

Reviewer #3:

Q13: The authors used a static assay to determine adhesion to polystyrene showing that Awp2 mediates binding to plastic in a time dependent manner. It is known that flow conditions might change binding of fungi to plastic as seen in catheters. Therefore, it would be interesting to see the contribution of Awp2 to adhesion under flow conditions.

A13: Thanks a lot to the reviewer for the valuable suggestion. Given the complexity of the effects of Epa- and Awp-like cell wall proteins to the biology of *C. glabrata*, this will be an important issue for future work. Clearly, the scope of this manuscript is the presentation of the diversity of Awp1-related adhesins in terms of function despite common structural architecture.
